# Study on the Complex Band Structure and Auxetic Behavior of Fractal Re-Entrant Honeycomb Metamaterials

**DOI:** 10.3390/ma18245695

**Published:** 2025-12-18

**Authors:** Jingru Li, Siyu Chen, Wei Lin, Yuzhang Lin

**Affiliations:** 1 School of Mechanical and Electrical Engineering, Hainan University, Haikou 570228, China; 23220854060025@hainanu.edu.cn (S.C.); 23210828010003@hainanu.edu.cn (Y.L.); 2 Qingdao Innovation and Development Center, Harbin Engineering University, Qingdao 266400, China; linwei23@hrbeu.edu.cn

**Keywords:** fractal metamaterials, attenuation diagram, band gaps, negative Poisson’s ratio, multiple functional design

## Abstract

**Highlights:**

**What are the main findings?**

- Higher-order re-entrant honeycomb MM is proposed using multi-level features.
- The complex band structure was systematically investigated.
- The auxetic behavior is demonstrated and correlated with structural hierarchy.

**What are the implications of the main findings?**

- The results provide insights into coupling band structures with auxetic mechanisms.
- The design strategy supports the development of multifunctional metamaterials.
- The study offers guidance for acoustic and mechanical metamaterial design.

**Abstract:**

In order to break the limitation of metamaterials used in the vibration and sound reduction field, this work designed a two-dimensional metamaterial based on the re-entrant honeycomb lattice and using the fractal technique. The first, second, and third-order fractal re-entrant honeycomb metamaterials are analyzed, respectively, within the established numerical models responsible for predicting the effective Poisson’s ratio, the real band structure, and the attenuation diagram. The effects of the fractal order, fractal ratio, and geometrical characteristics on these multiple functionalities are investigated simultaneously. Through adjusting the proposed fractal metamaterials, the results show that the transformation of auxetic performance, the number and location of multiple stop bands, the attenuation level inside the stop bands, and the wave decaying directionality can be flexibly tuned. This demonstrates that the compatibility of mechanical features and wave motion characteristics is successfully achieved in the present work. It provides a theoretical and technical basis for the development of multi-functional design methods of metamaterials in solving engineering problems.

## 1. Introduction

Metamaterials can be seen as representative artificial structures, which are able to possess extraordinary properties that natural materials do not possess. The innovative properties attract increasing attention from researchers [1,2]. Various categories of metamaterials, including thermal metamaterials [3,4], elastic metamaterials [5,6], mechanical metamaterials [7,8], acoustic metamaterials [9,10], and so on, have been developed to control wave energy concerned with different research fields. Among them, it is essential to design elastic metamaterials according to the characteristics of the band gaps. These gaps refer to specific frequency ranges where elastic or acoustic waves are fully forbidden to propagate. This facilitates the developments in a wide range of engineering applications, including vibration isolators [11,12,13], wave filters [14,15,16], sound insulation [17,18,19], wave guides [20,21,22], and energy harvesting [23,24,25].

The mechanisms to induce band gaps were found to be Bragg scattering [26], local resonance [27] and the inertial amplification [28], and the unique characteristics can be detected via the Bloch theory, based on which various analytical and numerical methods have been proposed, such as the transfer matrix method (TMM) [29], the spectral element method (SEM) [30], the plane wave expansion method (PWE) [31], the multiple scattering theory (MST) [32] and the finite element method (FEM) [33]. To gain more flexibility of band gaps, researchers began to apply multi-field coupling media [34], reconfigurable mechanisms [35], multi-level [6], and self-similarity technique [36] to design elastic metamaterials. Among them, the fractal self-similar fractal structures gradually become popular in the biological and mechanical territories [37,38] and have attracted widespread attention in controlling band gaps owing to the ability of generating low and multiple stop bands. Motivated by the fractal idea, the fractal design was introduced to the porous phononic crystal to study the band structures in Sierpinski triangle fractal porous phononic crystals (FPPCs) and clarify the effect of fractal hierarchy on the band structures [39]. Cheng et al. [40] proposed a hybrid metamaterial subjected to second-order fractal and series fusion, showing a complete band gap below 500 Hz, and elucidated the band gap properties in terms of band structure and group velocity with respect to different fractal levels. Focusing on the seismic waves, a broadband seismic metamaterial based on H-shaped fractal pillars was designed by Du et al. [41]. In this work, a comparative study on the band structure of seismic surface waves shows that a new level of fractal structure can create a new band gap, widen the total band gaps, and shift the same band gap towards lower frequencies. Furthermore, a honeycomb lattice was combined with fractal theory to isolate vibration and noise via analyzing the energy band structure and vibration transmission characteristics of different order fractal metamaterials [42]. Recently, Yang et al. [43] aimed to explore the influences of the position and filling ratio of internal fillers on the band structure of fractal metamaterials, and the results demonstrates that core filling at the central positions can produce significant effects on the formation and widening of low-frequency band gaps, while filling at corner edges predominantly affects mid-frequency band gaps, and filling at edge centers effectively opens and broadens high-frequency band gaps. However, the existing research focused on the fractal metamaterials do not address the complex band structure, only providing the real band structure, which is not able to qualitatively estimate the decaying level inside the stop bands, clearly showing a gap in the state-of-the-art for the fractal metamaterials design. Therefore, the present work aims to fill this gap. Generally, the complex band structure can be examined via the ***k***(*ω*) approach that allows one solve the eigenvalue problem for complex wave vectors as the frequencies are given [44,45,46]. In this way, the present work can precisely describe the characteristics of spatial attenuation caused by the evanescent waves through the fractal metamaterials.

Moreover, in the pursuit of innovative mechanical behavior, the artificial cells possessing a negative Poisson’s ratio (NPR) have attracted significant research interest in mechanical metamaterials, which was first designed and fabricated through a re-entrant foam [47] and then developed to the kirigami and origami cells [48,49,50]. Although this field has developed rapidly in recent years, its theoretical foundation was established decades ago. In 1982, the mechanical mechanism of the first two-dimensional re-entrant honeycomb structure was solved and analyzed [51], which theoretically proved the feasibility of inducing auxeticity through geometry. Later, this concept was extended to thermodynamic molecular systems. For example, a Monte Carlo simulation study in 1987 on two-dimensional hard cyclic hexamers showed that the densest phase of these molecules exhibits a stable negative Poisson’s ratio and elastic isotropy [52]. Subsequently, in 1989, analytical solutions for hexagonal molecules on a triangular lattice revealed that Poisson’s ratio could be flexibly tuned from strongly auxetic to non-auxetic values depending on the hexamer density [53]. After that, the term “Auxetics” was formally introduced by Evans et al. [54], clarifying the core counterintuitive feature of these materials: lateral expansion (or contraction) occurs under tension (or compression). From a theoretical perspective, it is also crucial that the isotropic bounds of Poisson’s ratio in 2D structures (−1 < ν < 1) are distinct from those in 3D cases (−1 < ν < 0.5), which provides a broader design space for planar metamaterials [53,55,56,57,58]. For anisotropic materials, the upper and lower bounds of Poisson’s ratio can be extremely large in theory. Accompanying these theoretical advancements, the first thematic issue [59] on auxetic materials was published in 2005, initiating a continuously updated knowledge base for auxetic materials and mechanical metamaterials. This auxetic behavior has been applied in tremendously promising applications such as energy absorption [60,61,62], counterintuitive deformation [63], and indentation resistance [64]. Recent studies also indicate that unavoidable defects and imperfections in real materials can significantly affect their mechanical responses. As early as 2014 and 2015, studies were conducted to discuss the impact of defects on antichiral structures and the translational disorder problem in hexachiral auxetic structures [65,66], emphasizing that structural imperfection is a key factor in practical applications. Unfortunately, limited researchers have combined the auxetic property with the fractal design for a thorough investigation of the complex band structure, which is a key consideration in structural and engineering applications. In order to make use of the abnormal mechanical and wave characteristics simultaneously, this paper adopts the re-entrant cell as the base configuration that is expected to be applied within the fractal technique. With respect to varying geometrical features and fractal order, a comprehensive study is conducted via assessing the effective Poisson’s ratio, the real band structure and the attenuation diagram, facilitating the development of fractal metamaterials with multiple functionalities, such as the tunable vibration isolators and composite sound absorbers focusing on low and wide frequency performance.

The remaining part of this work is organized as follows. Section 2 introduces the design of the proposed fractal metamaterial together with the methods to calculate the complex band structure and the effective negative Poisson’s ratio. The auxetic property is predicted and tuned in Section 3, and the propagation and attenuation characteristics are analyzed by combining the real band structure, the attenuation diagram, and the decaying directionality. The parametric analysis to verify the tunability of the fractal metamaterial on the complex band structure is also conducted. Finally, the main conclusions are summarized.

## 2. Model and Methods

### 2.1. Fractal Re-Entrant Honeycomb Metamaterial Design

**Figure 1:**
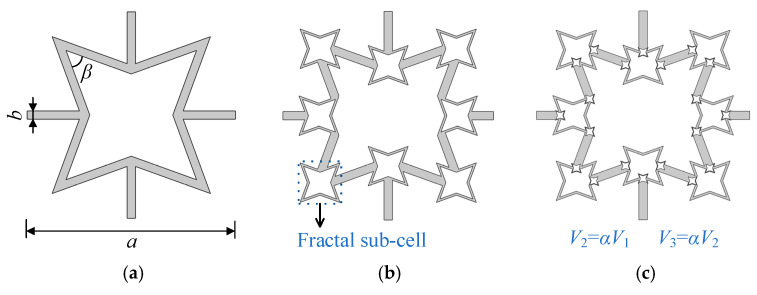
Design and models of fractal re-entrant metamaterials for different orders: (**a**) the first order; (**b**) the second order; (**c**) the third order.

In this work, the re-entrant honeycomb cell, which is illustrated in Figure 1a, serves as the foundational element for creating fractal metamaterials, whose geometrical features can be captured by the thickness of frame *b*, the angle *β*, and the unit cell length *a*. Inspired by this base configuration, higher levels or higher orders of fractal lattices are developed, as shown in Figure 1b,c, the generation of the higher-order fractal metamaterial follows a rigorous recursive algorithm. For an *n*-th order structure (*n* ≥ 2), the fractal sub-cells are generated by scaling down the (*n* − 1)-th order parameters, where both the unit cell length *a* and wall thickness *b* obey the scaling law defined by the fractal ratio α: $a_{n}=\alpha \cdot a_{n-1},\;b_{n}=\alpha \cdot b_{n-1}$, i.e., at each increased order, the relation between the volume of the newly generated sub-cell *V_n_* and that of the original cell *V_n−_*_1_ satisfies that: *V_n_* = *αV_n−_*_1_, meanwhile the lattice constant *a* remains unchanged. These sub-cells are embedded into the specific nodes of the parent structure (the previous-order structure). Regarding spatial positioning, the sub-cells located at the top-left and top-center are aligned by coinciding their geometric centers with the geometric centers of the parent nodes. For the left-side sub-cell, a strict topological constraint is imposed to ensure the geometric continuity with neither overlap nor gaps. For higher orders (*n* ≥ 3), this embedding process is repeated recursively at the newly formed connection nodes.

The proposed fractal metamaterial is generated through an iterative substitution mechanism, in which local segments of the (*n* − 1)-th order structure are replaced by scaled fractal sub-cells. As a result, the evolution of physical quantities—particularly the structural mass—follows a recursive scaling law. The total mass *M_n_* of the n-th order structure is governed by the net mass change introduced at each iteration:

(1) $$M_{n}=M_{n-1}+N_{sites}^{\left(n\right)}\cdot \left(m_{sub}^{\left(n\right)}-m_{cut}^{\left(n\right)}\right)$$

Here, *M_n_*_−1_ denotes the mass of the previous-order structure, and $N_{\mathrm{sites}}^{\left(n\right)}$ is the number of substitution locations at order n. The term $m_{\mathrm{sub}}^{\left(n\right)}-m_{\mathrm{cut}}^{\left(n\right)}$ represents the net mass gain at each substitution, obtained from the difference between the mass of the inserted fractal sub-cell and the mass of the removed base-segment.

The proposed metamaterials are termed as the first, second, and third-order fractal re-entrant honeycomb metamaterials (FRHMs), respectively. Following this, the complex band structure consisting of the real band diagram and attenuation diagram can be investigated based on the first three order FRHMs. Additionally, to predict the auxetic behavior induced by the fractal technique at the same time, the fractal metastructure composed of finite corresponding unit cells is also taken into account for conducting static analysis.

### 2.2. Complex Wave Dispersion Relation

The real band structure is obtained via the *ω*(***k***) approach, while the attenuation diagram is derived through the ***k***(*ω*) approach, whose methods are all developed based on the Bloch theorem. According to this theory, the point in the unit cell can be expressed as the following when the elastic waves propagate through the x-o-y plane:

(2) $$R_{n}=n_{1}a_{1}+n_{2}a_{2}$$

where ***R****_n_* denotes the lattice vector while ***a***_1_ and ***a***_2_ represents the base vectors of the primitive lattice. As the present work considers square lattices sharing the same lattice constant, the basis vectors can be formulated as:

(3) $$\begin{array}{l} a_{1}=(a,0)\\ a_{2}=(0,a) \end{array}$$

Based on Equation (3), the set of reciprocal lattice basis vectors ***b***_1_ and ***b***_2_ can be easily obtained owing to the underlying transformation relationship with the primitive lattice basis vectors:

(4) $$a_{i}\cdot b_{j}=\delta_{ij}$$

Ignoring the external work, the harmonic wave equation follows the form:

(5) $$\begin{array}{l} \rho \omega^{2}u+\left(\lambda +2G\right)\frac{\partial^{2}u}{\partial x^{2}}+\left(\lambda +2G\right)\frac{\partial^{2}v}{\partial y\partial x}+G\frac{\partial^{2}u}{\partial y^{2}}=0\\ \rho \omega^{2}v+\left(\lambda +2G\right)\frac{\partial^{2}v}{\partial y^{2}}+\left(\lambda +2G\right)\frac{\partial^{2}u}{\partial x\partial y}+G\frac{\partial^{2}v}{\partial x^{2}}=0 \end{array}$$

The Bloch theory requires the displacement field (*u,v*) satisfy:

(6) $$\begin{array}{l} u\left(r,k,t\right)=U\left(r,k\right)e^{-ik\cdot r}e^{i\omega t}\\ v\left(r,k,t\right)=V\left(r,k\right)e^{-ik\cdot r}e^{i\omega t} \end{array}$$

**Figure 2:**
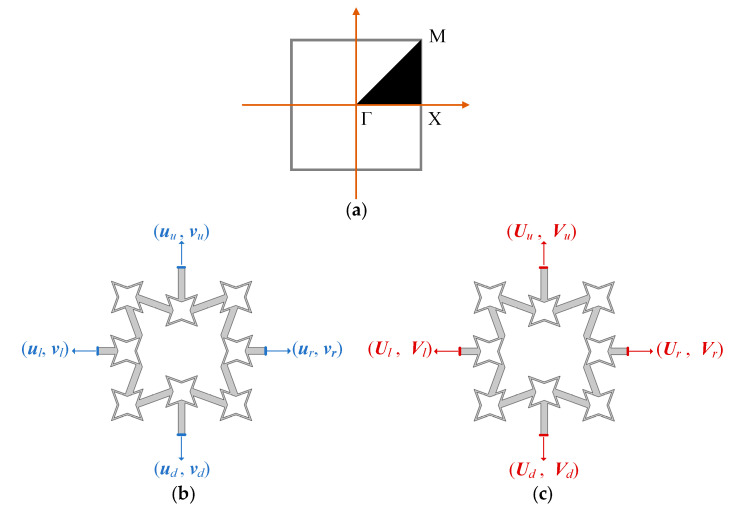
(**a**) The IBZ of the square lattice and the division of the displacement field according to the periodic boundary condition described by: (**b**) the *ω*(***k***) approach and (**c**) the ***k***(*ω*) approach.

The combination of Equations (5) and (6) leads to two approaches to examine the dynamic properties of the wave motion. One typical approach is forcing Equation (5) to be solved in a ω(***k***) form where u and v are regarded as displacement variables for acquiring the eigenvalue of frequency ω with respect to a given Bloch wave vector ***k***. Figure 2a gives the irreducible Brillouin zone (IBZ) characterized by a black region. The real band structure can be obtained via scanning the wave vectors through the boundary of the IBZ. In particular, when adopting the FEM method, the discrete formulation of Equation (5) is consistent with the common FEM but applied with a periodic boundary condition that can be expressed as:

(7) $$\begin{array}{l} u_{r}=u_{l}e^{-ik_{x}a}\\ u_{u}=u_{d}e^{-ik_{y}a} \end{array}$$

(8) $$\begin{array}{l} v_{r}=v_{l}e^{-ik_{x}a}\\ v_{u}=v_{d}e^{-ik_{y}a} \end{array}$$

where the physical meaning of the subscripts illustrates the location of the displacement component in the unit cell, which can be seen from Figure 2b via the second-order FRHM.

In contrast, in another approach—the ***k****(ω)* approach, the frequencies are known from Equation (5), and the wave vector ***k*** is calculated and generally expressed by a complex solution. The FEM model is not the traditional form since ***U*(*r,k*)** and ***V*(*r,k*)** are transformed to the displacement variables. Hence, the periodic boundary conditions are reformulated as following and are illustrated in Figure 2c:

(9) $$\begin{array}{l} U_{r}=U_{l}\\ U_{u}=U_{d} \end{array}$$

(10) $$\begin{array}{l} V_{r}=V_{l}\\ V_{u}=V_{d} \end{array}$$

Applying Equations (5), (6), (9) and (10) to the coefficient form partial differential equation (PDE) module of COMSOL 6.3, the FEM model can be established to solve the complex wave vectors. The smallest imaginary part of the complex wave number is defined as the attenuation constant δ=minIm(ka)/π, which is responsible for examining the decaying level through space.

### 2.3. The Auxetic Properties Analysis

The effective Poisson’s ratio is predicted via FEM modelling of finite fractal metastructures for the first three orders. Each of them is composed of 16 × 16 corresponding unit cells. Obviously, the fundamental lattice can possess a negative effective Poisson’s ratio; however, the effects caused by the addition of fractal components remain unclear.

**Figure 3:**
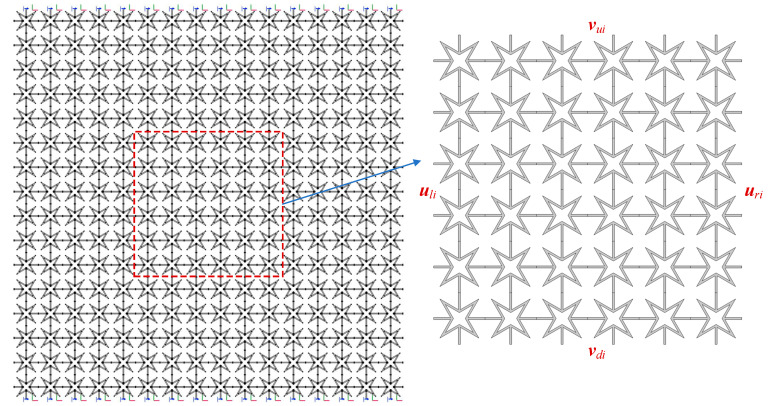
The descriptions on the FEM modelling for computing the effective Poisson’s ratio.

To derive the effective Poisson’s ratio, the uniaxial tension is exerted at the vertical ends of the considered finite metastructures. Meanwhile, the center region, including 6 × 6 unit cells, highlighted by the red rectangle, is regarded as the domain of interest for calculating tensile and transverse strains. This aims to eliminate the effects of the outer boundary of the finite frame on the results. Figure 3 illustrates the FEM modelling where the first-order fractal metastructure is selected as an example for brevity purposes. Based on the displacement field derived from this interested domain, the effective Poisson’s ratio $\bar{\nu }$ can be calculated by the following:

(11) $$\bar{\nu }=-\frac{({\bar{u}}_{ri}-{\bar{u}}_{li})/6a}{({\bar{v}}_{ui}-{\bar{v}}_{di})/6a}$$

where the subscripts “*ri*”, “*li*”, “*ui*”, and “*di*” represent the boundary of the domain and $\bar{u}$ $\vec{v}$ are the average displacements over the boundary lines.

## 3. Results and Discussions

### 3.1. The Auxetic Behavior Analysis with Fractal Technique

As described in the above sections, the first-order fractal metamaterial is derived from the negative Poisson’s ratio unit cell, which can exhibit auxetic behavior. When the base unit is applied with the fractal design strategy, the generated multi-level features lay down unknown mechanisms to tune the effective Poisson’s ratio in this manner. To this end, this subsection aims to demonstrate the adjustable ability of controlling the effective Poisson’s ratio combined with self-similarity. Specifically, the first three orders of re-entrant honeycomb shapes are all taken into account. Simulations are performed to predict the auxetic behavior.

The geometrical and material parameters of the FRHMs are presented in Table 1 and Table 2, respectively. In this work, the material belongs to the high polymer material. It is noted that these values remain unchanged unless stated. Based on these properties, the finite metastructures consisting of corresponding order unit cells are constructed, respectively, to derive the effective Poisson’s ratio. In this work, the fractal ratio *α* is assumed to be 1/4, 1/5, and 1/6, respectively. It is noted that the mesh convergence is assured in advance.

**Figure 4:**
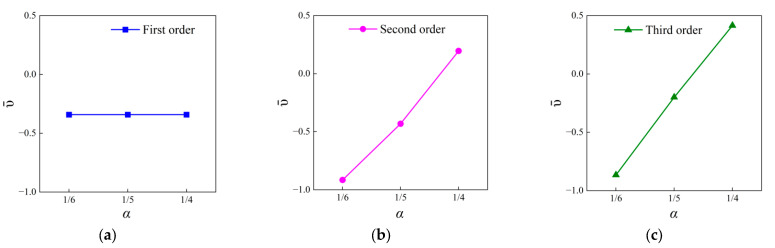
The tuning characteristics of auxetic behavior versus α for the first three order FRHMs: (**a**) the first order; (**b**) the second order, and (**c**) the third order.

**Figure 5:**
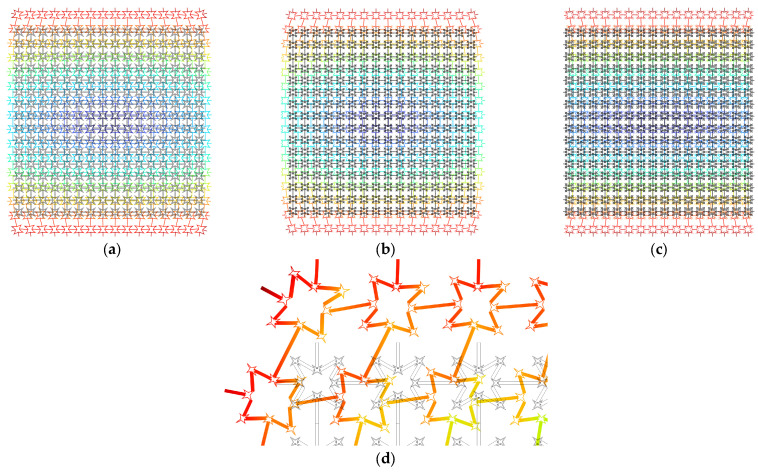
The deformed state of FRHMs within uniaxial tension: (**a**) the first order; (**b**) the second order (*α* = 1/5); (**c**) the second order (*α* = 1/4); (**d**) the third order (*α* = 1/6).

The results for the first three orders are shown in Figure 4. It is noted that the first-order FRHM is independent of the fractal ratio *α* and thus the values of $\bar{\nu }$ keep still, resulting in a horizontal line from Figure 4a. Its value reaches −0.342, which obviously reflects the intrinsic property of the negative Poisson’s ratio. The corresponding deformation is illustrated in Figure 5a. The auxetic behavior can be clearly observed here. Specifically, the finite structure is stretched along the x-direction when subjected to the uniaxial tension from the y-direction. In contrast, as seen from Figure 4b, owing to addiction of smaller self-similar parts, the value $\bar{\nu }$ further decreases to −0.915 compared to the first order when *α* is equal to 1/6, indicating that the auxetic property can be enhanced via the fractal technique. However, the value of the effective Poisson’s ratio increases with respect to increasing *α*. They are calculated as −0.431 and 0.196, corresponding to *α* = 1/5 and *α* = 1/4 of the second order. This is because the fractal technique breaks the re-entrant characteristics of the vertices in the horizontal direction. It transforms them into convex angles. However, the structure still inherits the intrinsic negative Poisson’s ratio along the vertical direction. If the value of *α* is small, the influences caused by the remaining re-entrant feature surpass that induced by the transformed vertex, intensifying the negative Poisson’s ratio performance. Nevertheless, as *α* increases, the geometrical attribute of the second-order addition presented in the horizontal direction becomes more prominent, which leads to the generation of a positive effective Poisson’s ratio. Figure 5b,c show the deformed metastructures exerted by the same load in the cases of *α* = 1/5 and *α* = 1/4, clearly reflecting the phenomenon caused by negative and positive effective Poisson’s ratios, respectively.

This explanation can further be verified from the results derived from the third-order FRHM. From Figure 4c, one can observe the values of $\bar{\nu }$ are −0.866, −0.199, and 0.415 among the considered cases, rising more rapidly compared to the second-order FRHM. The deformed configuration corresponding to *α* = 1/6 is also given in Figure 5d, which is in accordance with the results of the effective Poisson’s ratio. As illustrated above, the proposed FRHM in this paper can undoubtedly enhance the flexibility of controlling the auxetic behavior. Moreover, this tunability is helpful to realize “zero Poisson’s ratio” property via cascading the same order FRHMs characterized by different fractal ratios, making it possible to acquire low and broad frequency vibration and sound reductions, while meeting the load-bearing requirements.

### 3.2. Complex Band Structure of FRHM

In contrast to previous studies of fractal metamaterials which only focus on the real band diagrams, this subsection aims to provide the complex band structure consisting of real and imaginary parts of dispersion relations, which is able to examine the frequency range, the location as well as the decaying level of stop bands simultaneously, further gaining a complete view on the unique spatial attenuation as the self-similarity is introduced.

#### 3.2.1. Real Band Diagram

First, the real band structure is calculated regarding wave vectors assigned at the boundary of the IBZ shown in Figure 2a, whose objective is to observe the location and size of complete band gaps while investigating the influences caused by the fractal order. The geometrical and material characteristics are also derived from Table 1 and Table 2. The FEM model is established in the Solid Mechanics Module of Comsol within eigenvalue analysis. In this subsection, the fractal ratio *α* is fixed at 1/5 at first. In order to differentiate the location of band diagrams, the resultant frequencies are normalized as follows:

(12) $$\Omega =\omega a/2\pi c_{t}$$

where *c_t_* denotes the speed of transverse waves.

**Figure 6:**
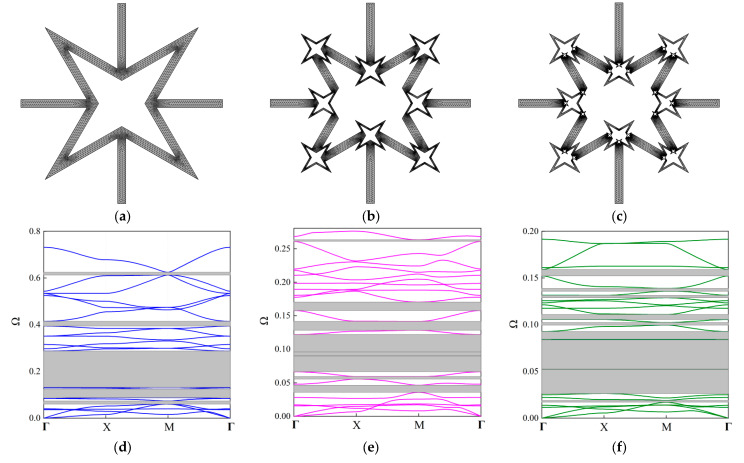
The established FEM models for (**a**) first-order, (**b**) second-order, and (**c**) third-order unit cells, and the real band diagrams of FRHMs for the (**d**) first order, (**e**) second order, and (**f**) third order.

The first 20 branches are taken into account to constitute the real band diagram and shown in Figure 6d–f for the first three orders. From Figure 6, the FRHM exhibits multiple complete stop bands shaded by gray rectangles, where several extremely narrow stop bands are ignored. Obviously, owing to the fractal partitions, the real band diagrams are forced to move towards lower frequencies. With the increasing of the fractal order, the number of complete stop bands also increases. As a result, the first 20 branches’ dispersion curves exhibit 5, 8, and 9 complete stop bands for the first, second, and third-order FRHM, respectively. Concentrated on the first 20 branches, the largest relative bandwidth is 148%, which is found from the third-order FRHM, indicating the potential of lowering and broadening band gaps via applying the fractal technique. Table 3 summarizes the boundaries of the complete stop bands for each order FRHM.

**Figure 7:**
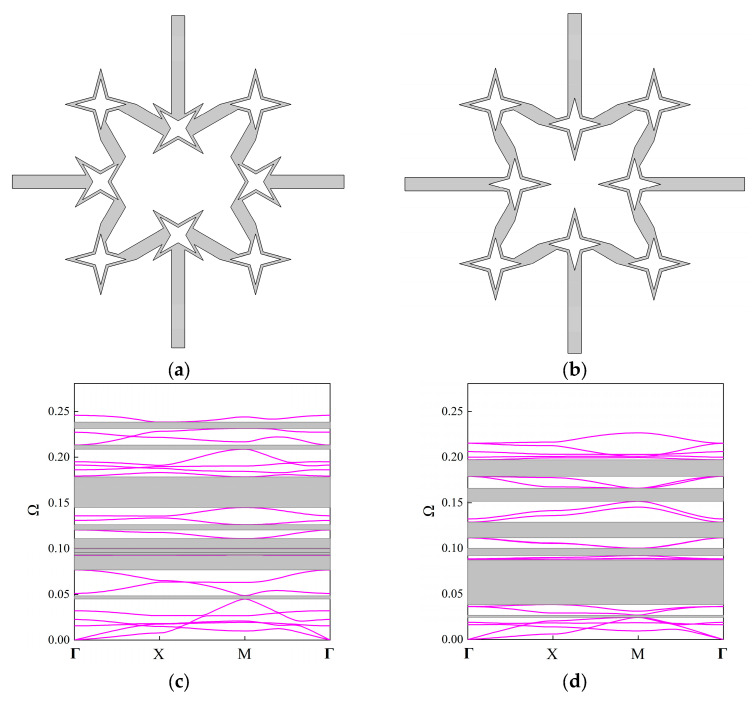
The new configurations obtained by rotating the fractal parts: (**a**) Configuration V-I; (**b**) Configuration V-II; and their corresponding real band diagrams in (**c**,**d**).

In order to show the flexibility of the propose FRHM, the fractal parts are rotated 45 degrees to obtain new configurations, which are termed Configuration V-I and V-II, respectively. The second-order FRHM is selected for brevity purposes, and the detail information is shown in Figure 7a,b. Figure 7c,d presents the corresponding band structures. As can be seen, after rotation, the entire band structures are lowered, and with respect to more rotation parts, the drop becomes more intensive. The dispersion curves at lower frequencies vary slightly compared to Figure 6b, but those at higher frequencies exhibit distinctive features as the fractal parts are responsible for determining the higher-order branches. As a result, the bandwidth and location of multiple stop bands can be flexibly tuned through rotating the fractal parts, verifying the design potential of the proposed FRHM. In particular, it can be seen that Configuration V-II is able to possess better low-frequency performance among the considered cases owing to the ultra-broad complete stop band appearing around 0.05.

To further study the effects of the fractal design on the real band diagrams, an analysis for the second-order FRHM is conducted. The fractal ratio *α*, the thickness *b,* and the angle of the re-entrant honeycomb shape *β* are taken into account to determine the geometrical properties of FRHM, while keeping other parameters constant.

**Figure 8:**
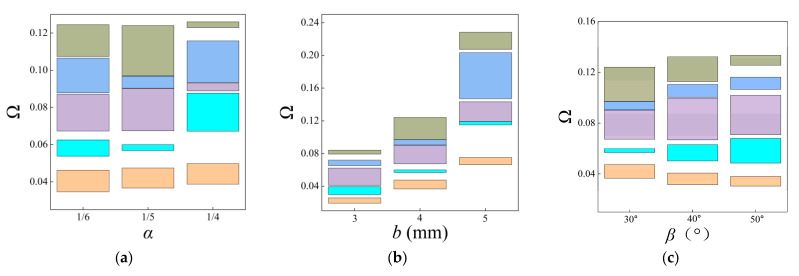
The adjustability on multiple stop bands via controlling the geometrical features of the second-order FRHM: (**a**) the fractal ratio *α*; (**b**) the thickness *b*; (**c**) the angle *β*.

Firstly, the ratio *α* is varied from 1/5 to 1/4 and 1/6 separately, and the comparison of the first fifth complete stop bands among the three cases is presented in Figure 8a. One can notice that the stop bands move to a higher location as *α* increases, whose bandwidth can be broadened and narrowed by changing the fractal ratio. Secondly, other parameters remain still; the thickness *b* is considered to be 3 mm, 4 mm, and 5 mm, respectively, in which case the locations of complete stop bands are computed and provided in Figure 8b. As can be seen, the bigger value of the thickness *b* is able to lift the band diagram and broaden the total bandwidth. Finally, Figure 8c shows the results with respect to different angles *β*. The trend is different from that observed in Figure 8a,b. The lower stop bands continue to shift towards lower locations, and higher stop bands move towards higher positions as *β* increases. In sum, multiple complete stop bands can be flexibly adjusted by modifying the fractal design parameters.

#### 3.2.2. The Attenuation Diagrams

In contrast to the above investigations on the real band structures, this subsection aims to examine the attenuation level inside the stop bands based on the fractal unit cell. It is achieved by calculating the imaginary parts of complex wave vectors with respect to given frequencies. As stated before, this approach is available for acquiring attenuation characteristics of elastic waves in an arbitrary direction. The attenuation constant *δ* of elastic waves propagating through the ΓX direction is computed first, which can estimate the degree of suppressing wave propagation inside the stop bands. The parameters of the unit cells with the first three orders of the FRHM follow the set in Table 1, and the covering frequency ranges for each order concerned with the ΓX direction are assumed as (0, 0.36), (0, 0.15), and (0, 0.12), respectively. Figure 8 provides the results of the attenuation diagrams as well as the comparisons to their corresponding real band structures, scanning the ΓX wave space.

**Figure 9:**
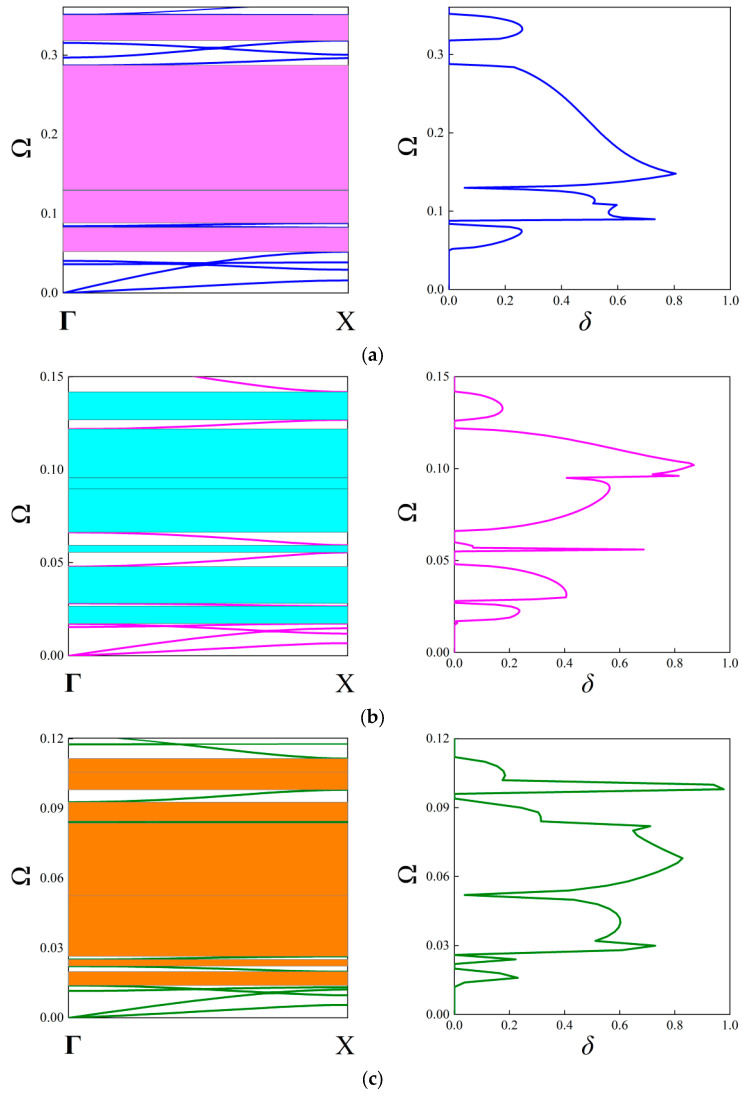
The attenuation diagrams along the ΓX direction with the comparison to the real band structure for the (**a**) first-order, (**b**) second-order, and (**c**) third-order FRHMs.

From each attenuation diagram in Figure 9, it can be seen that the attenuation regions defined by *δ* match well with the directional stop bands derived from real band diagrams, validating the correctness of the results of complex band structures. Additionally, the attenuation diagrams can distinctly reflect the decaying level of each stop band. Over the first 10th branches of the dispersion curves, the average constant is calculated as 0.34, 0.3, 0.405 for the first three orders, whereas the largest amplitude is 0.8, 0.87, and 0.98, which shows an enhancement using higher fractal order. Furthermore, the mechanism for inducing directional stop bands can be transformed by applying the fractal technique. For example, the first stop band shown in Figure 9a is caused by the Bragg scattering, but that in Figure 9c is generated owing to the local resonance, realizing the transition of band gap mechanisms. Moreover, as the complex band structure can offer the directionality of wave attenuation characteristics across the entire in-plane space, the following gives the attenuation constants from all directions at the specific frequencies in the cases of the considered FRHMs.

**Figure 10:**
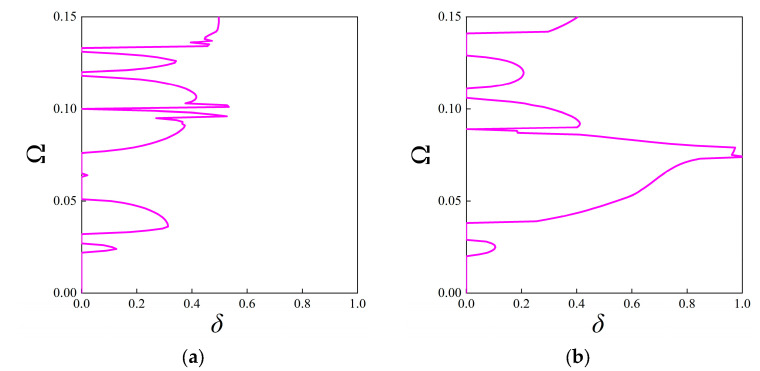
The attenuation diagrams along the ΓX direction for (**a**) Configuration V-I and (**b**) Configuration V-II.

Moreover, Configurations V-I and V-II are also taken into account, and Figure 10 presents the attenuation diagrams along the ΓX direction. One can notice that the Configuration V-I that contains both rotated fractal components and original fractal components exhibits smaller amplitudes through the entire interested frequency range compared to that from Figure 9b. However, the peak value offered by Configuration V-II is the largest among the considered cases, and the second directional stop band exhibits wide and strong attenuation characteristics, which are conducive to isolating vibration and sound. The average constant for the configuration in Figure 9b, configurations V-I and V-II, is calculated as 0.2639, 0.1926, and 0.2869, respectively, indicating the superior performance of Configuration V-II. Nevertheless, the original configuration can possess better attenuation performance at lower frequencies (below 0.05), which also validates the tunability of the proposed FRHM.

#### 3.2.3. Directionality of Elastic Wave Decaying

**Figure 11:**
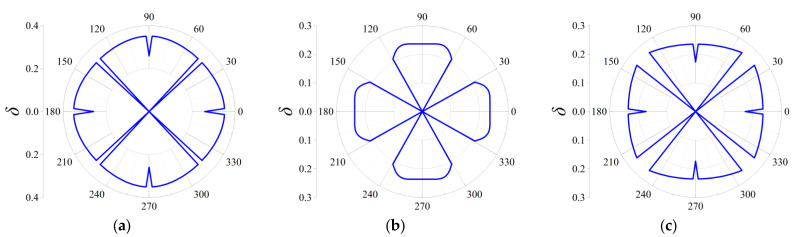
Directional attenuation constants of the first three orders of FRHMs in Case I: (**a**) the first order; (**b**) the second order; (**c**) the third order.

**Figure 12:**
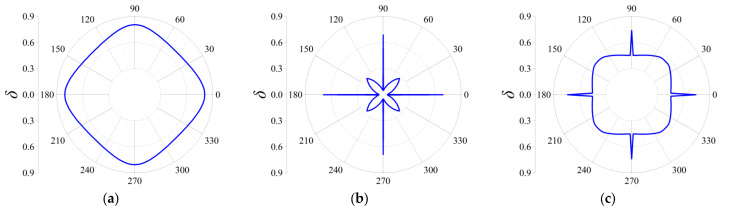
Directional attenuation constants of the first three orders of FRHMs in Case II: (**a**) the first order; (**b**) the second order; (**c**) the third order.

**Figure 13:**
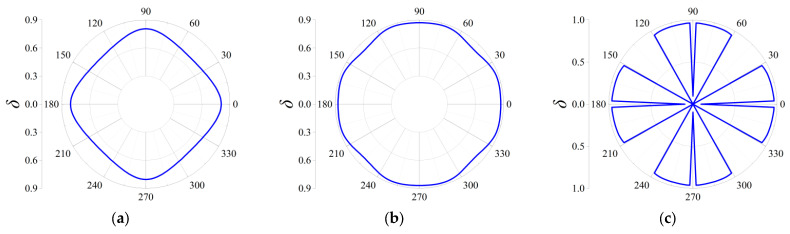
Directional attenuation constants of the first three orders of FRHMs in Case III: (**a**) the first order; (**b**) the second order; (**c**) the third order.

Three cases termed as Case I, Case II and Case III are taken into consideration to discover the influences on the attenuation directionality of the FRHMs, and in the former two cases, the computed frequencies correspond to the locations of attenuation peaks of the first and third ΓX directional stop bands for each order FRHM, while in the last case, the considered frequencies denote that of the largest attenuation constant over the first 10th branches. Therefore, in Case I, the specific frequencies are 0.074, 0.023, and 0.016 for the first, second, and third-order FRHMs; these frequencies are 0.148, 0.056, and 0.03 in Case II and 0.148, 0.102, and 0.098 in Case III. Owing to the highest-level symmetry characteristics that various order lattices possess, the complete attenuation profile can be obtained by calculating the attenuation constants only within the range of wave propagation *θ* from 0 to π/4. The results are provided in Figure 11, Figure 12 and Figure 13 for Case I, Case II, and Case III.

In Case I, the selected frequency for the first order is around the boundary of the first complete stop band, and thus the wave attenuation can be merely observed from all directions, which is able to be observed from Figure 11a. Nevertheless, the other two frequencies are located at the directional stop band but are away from the first complete stop band, and correspondingly, as seen from Figure 11b,c, the attenuation constants show a more intensive anisotropy degree with respect to propagating angle *θ*, and even the wave attenuation ability can be found invalid across some angles.

Similarly, as shown in Figure 12a–c, elastic waves propagating from all directions can be blocked, since the computed frequencies are all located in the corresponding complete stop bands. But there still exist differences among the three orders in Case II. It is clearly seen that the second-order FRHM exhibits the strongest anisotropy level of wave attenuation, where the attenuation constant from the ΓX direction is still larger than that derived from other directions. However, this anisotropy degree is reduced as the considered frequency moves to a higher position, which can be verified by observing the directionality from Figure 13b, where comparable decaying level through arbitrary directions is acquired as a result. Meanwhile, regarding the third-order FRHM, the attenuation peak inside the directional stop band is outside the complete stop band, and thus arbitrary decaying ability disappears, as found from Figure 13c.

#### 3.2.4. The Parametric Analysis

To gain a comprehensive understanding of the attenuation characteristics of FRHMs, the second order with explicit fractal features is taken into account as a typical research object to investigate the effects of the fractal ratio *α*, the thickness *b*, and the angle of the re-entrant honeycomb shape *β* on the attenuation degree and the angular dependence.

**Figure 14:**
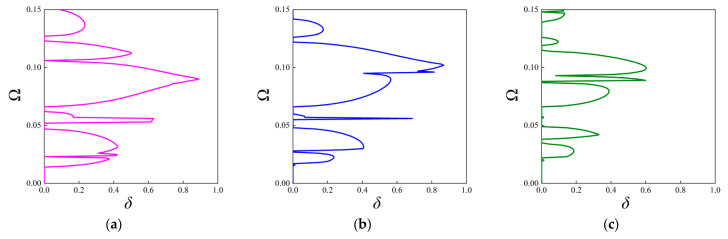
The attenuation diagrams along the ΓX direction with respect to different values of fractal ratio α: (**a**) α = 1/6; (**b**) α = 1/5, and (**c**) α = 1/4.

**Figure 15:**
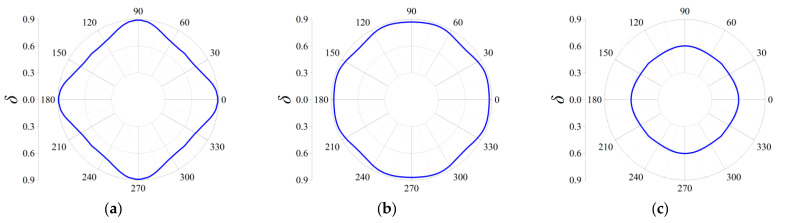
Directional attenuation constants with respect to different values of fractal ratio α: (**a**) α = 1/6, (**b**) α = 1/5, (**c**) α = 1/4 at specific frequencies.

The variations of these parameters are assumed to be consistent with the above discussion on the real band diagrams. Firstly, the fractal ratio *α* is assumed to be 1/6, 1/5, and 1/4, and in each case, the decaying level across the frequencies ranging from 0 to 0.15 is examined. The comparison is shown in Figure 14. As can be seen, different configurations determined by the fractal ratio exhibit distinctive attenuation diagrams. Obviously, the biggest partition adhered to the base re-entrant unit cell induce weakest attenuation degree, as shown in Figure 14c. The average attenuation constants for the FRHMs are computed as 0.31, 0.26, and 0.16, indicating that the smaller fractal ratio can enhance the decaying level, as well as showing the flexibility and efficiency to control the wave attenuation level inside the stop bands via using the fractal technique. In addition, the trend of the location of the attenuation diagrams is consistent with that found in real band diagrams of Section 3.2.1, i.e., decreased *α* can force the diagram move towards lower frequencies. The directionality of wave attenuation corresponding to the peak value across the interested frequency range is also illustrated in Figure 15. As a result, the computed frequencies are 0.09, 0.102, and 0.099.

From Figure 15, it can be seen that each directional stop band where the attenuation peak locates exhibit decaying capacity from all directions, i.e., these are all complete stop bands. The adjustment of the fractal ratio can manipulate the anisotropy degree that is aggravated within the smallest fractal ratio for the second-order FRHM. Additionally, the strength of attenuation degree is consistent with that found in Figure 14.

**Figure 16:**
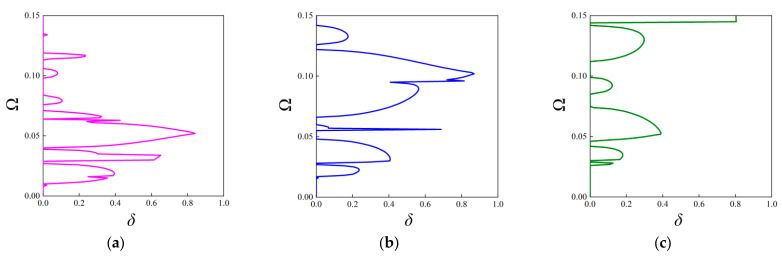
The attenuation diagrams along the ΓX direction with respect to different values of thickness *b*: (**a**) *b* = 3 mm; (**b**) *b* = 4 mm, and (**c**) *b* = 5 mm.

**Figure 17:**
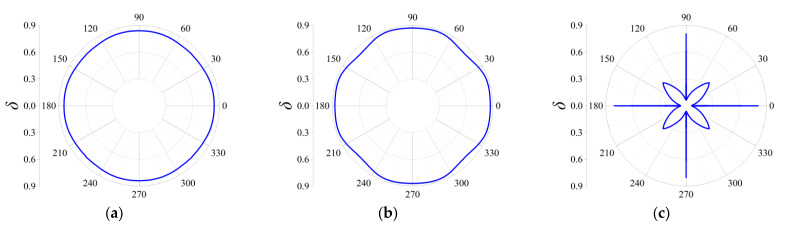
Directional attenuation constants with respect to different values of thickness *b*: (**a**) 3 mm, (**b**) 4 mm, and (**c**) 5 mm at specific frequencies.

Secondly, the thickness *b* is varied to observe the influences on the attenuation degree, and the results are compared in Figure 16. One can observe that the enhancement of the decaying level at lower frequencies can be achieved by adopting a smaller value of the thickness *b*. Consequently, the average attenuation constant for the considered cases is 0.17, 0.26, and 0.15, respectively. To detect the effects on attenuation directionality of elastic waves, the frequencies corresponding to the peak value are also taken into account, i.e., 0.052, 0.102, and 0.145. As found from Figure 17, a bigger value of *b* can aggravate the degree of attenuation anisotropy.

**Figure 18:**
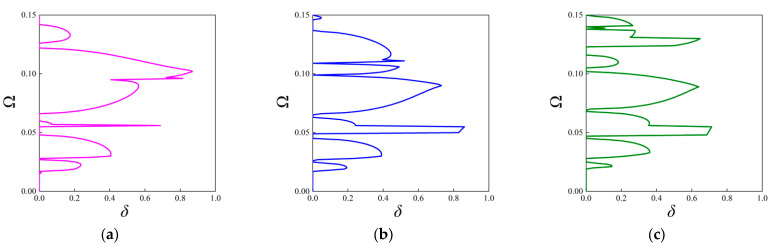
The attenuation diagrams along the ΓX direction with respect to different values of angle *β*: (**a**) *β* = 30°; (**b**) *β* = 40°, and (**c**) *β* = 50°.

**Figure 19:**
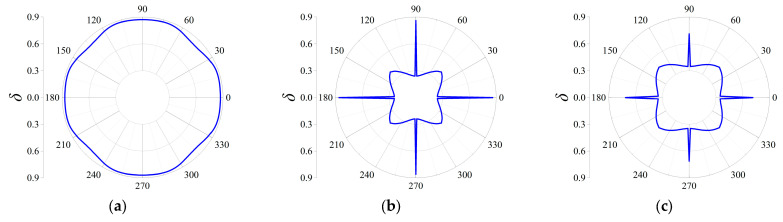
Directional attenuation constants with respect to different values of angle *β*: (**a**) 30°, (**b**) 40°, and (**c**) 50° at specific frequencies.

At last, this work investigates the effects of the angle of re-entrant base Configuration *β* on the attenuation diagram. Figure 18 shows the results of the directional stop band corresponding to the cases of *β* = 30°, 40°, and 50°. In accordant with the variations observed from real band structures, slight changes in attenuation region and level are occurring at lower frequencies, but visible differences can be found at higher frequencies when angle *β* varies. The average attenuation constant over the considered frequency range for each case is 0.26, 0.28, and 0.25, respectively. Furthermore, the peak value is observed around the frequencies of 0.102, 0.055, and 0.055, where the directionality of the attenuation diagram is examined and compared in Figure 19. The degree of attenuation anisotropy in Figure 19b can be comparable to that in Figure 19c, but they are all stronger compared to Figure 19a, showing the role of angle *β* for adjusting the wave directionality.

## 4. Conclusions

Inspired by the negative Poisson’s ratio structure used in engineering applications, higher orders of fractal re-entrant metamaterials are designed based on the fractal technique in this paper. To meet the requirements of multi-functional metamaterials, the auxetic behavior, the real band structure, and the attenuation diagram are systematically investigated at the same time. The results show that as the fractal order increases, the effective Poisson’s ratio can be flexibly adjusted from negative values to positive values via manipulating the fractal ratio. Due to the added fractal components, the real band structure is forced to move to lower frequencies, and the third-order FRHM can achieve the largest relative bandwidth. In addition, over the first 10th branches of dispersion curves, the average attenuation level inside the stop bands is found to be enhanced by adopting a higher fractal order. The directionality for prohibiting wave propagation is also dependent on the fractal order. Moreover, to gain a comprehensive understanding, the parametric analysis on the complex band structure regarding the second-order fractal metamaterial is also conducted in terms of varying fractal ratio, thickness, and base angle. With increasing fractal ratio, the stop bands are forced to shift towards higher positions, and the attenuation degree inside them worsens. As a result, the anisotropy degree is aggravated within the smallest fractal ratio for the second-order FRHM. Similarly, the enhancement of the decaying level at lower frequencies can be achieved by adopting a smaller thickness. In contrast, a bigger thickness can aggravate the degree of attenuation anisotropy. It is also found that by adjusting the base angle, the effects on the band gaps at higher frequencies surpass those at lower frequencies. Therefore, this study can be used to develop the multi-functional metamaterials in the vibration and sound reduction field that possess rich design freedoms.

## Figures and Tables

**Table 1:** Geometrical parameters used for constructing FRHMs.

Geometric Parameters	Thickness *b* (m)	Angle *β* (°)	Unit Cell Length *a* (m)	Fractal Ratio *α*
Values	0.004	30	0.1	1/5

**Table 2:** The material properties of the proposed FRHMs.

Material Properties	Young’s Modulus *E* (Pa)	Poisson’s Ratio *υ*	Density *ρ* (kg/m^3^)
Values	2.2 E9	0.394	1100

**Table 3:** The boundary of complete stop bands for each order FRHM.

Various Order FRHMs	The Bounding Frequencies of Considered Stop Bands								
	1st	2nd	3rd	4th	5th	6th	7th	8th	9th
1st order	(0.0602, 0.074)	(0.0881, 0.1247)	(0.1326, 0.2870)	(0.394, 0.4145)	(0.6131, 0.6246)	\	\	\	\
2nd order	(0.0358, 0.0464)	(0.0556, 0.0594)	(0.0664, 0.0898)	(0.0902, 0.0957)	(0.0958, 0.1219)	(0.1282, 0.1415)	(0.1577, 0.1704)	(0.2610, 0.2634)	\
3rd order	(0.0171, 0.0019)	(0.0264, 0.052)	(0.0525, 0.0837)	(0.0846, 0.0927)	(0.0996, 0.1023)	(0.1055, 0.1109)	(0.1289, 0.1314)	(0.1358, 0.1389)	(0.1523, 0.1592)

## Data Availability

The original contributions presented in this study are included in the article. Further inquiries can be directed to the corresponding author.

## References

[1] Fernandez-Corbaton I., Rockstuhl C., Ziemke P., Gumbsch P., Albiez A., Schwaiger R., Frenzel T., Kadic M., Wegener M. (2019). New Twists of 3D Chiral Metamaterials. Adv. Mater..

[2] Tan K.T., Huang H.H., Sun C.T. (2012). Optimizing the band gap of effective mass negativity in acoustic metamaterials. Appl. Phys. Lett..

[3] Jin P., Liu J., Xu L., Wang J., Ouyang X., Jiang J.H., Huang J. (2023). Tunable liquid–solid hybrid thermal metamaterials with a topology transition. Proc. Natl. Acad. Sci. USA.

[4] Wang Z., Zhu Z., Liu T., Hu R. (2022). Inverse design of thermal metamaterials with holey engineering strategy. J. Appl. Phys..

[5] Tomita S., Masutani T., Sato H. (2025). Locally resonant metamaterials damped by particles embedded through additive manufacturing. J. Sound. Vib..

[6] Cao L., Wu J., Zhang Z., Zhang C., Wan W., Bao J., Gao Y. (2024). Design optimization of elastic metamaterials with multilayered honeycomb structure by Kriging surrogate model and genetic algorithm. Struct. Multidiscip. Optim..

[7] Wang W.J., Yang H., Zhang W.M., Shang N., Ma L. (2025). Experimental study on the impact resistance of fill-enhanced mechanical metamaterials. Int. J. Mech. Sci..

[8] Liu Y., Dou S., Du Y., Liang R., Yue S., Zhao L., Liu F., Sun Z., Yang J. (2025). Enhanced broadband low-frequency performance of negative Poisson’s ratio metamaterials with added mass. Sci. Rep..

[9] Chong Y.B., Chen Z., Li Y., Lim K.M., Lee H.P. (2025). Acoustic metamaterial using hollowed star-shaped structure with slits. Appl. Acoust..

[10] Wang X., Xiao X., Han J., He Y. (2025). Honeycomb-cored hierarchical acoustic metamaterials: A synergistically coupled architecture for enhanced broadband sound absorption. Compos. Struct..

[11] D’Alessandro L., Ardito R., Braghin F., Corigliano A. (2019). Low frequency 3D ultra-wide vibration attenuation via elastic metamaterial. Sci. Rep..

[12] Chen Z., Sun S., Deng L., Yang J., Zhang S., Du H., Li W. (2022). Investigation of a new metamaterial magnetorheological elastomer isolator with tunable vibration bandgaps. Mech. Syst. Signal Process.

[13] Deng J., Yang J., Jiao S., Long X. (2023). Band-stop characteristics of a nonlinear anti-resonant vibration isolator for low-frequency applications. Int. J. Mech. Sci..

[14] D’Alessandro L., Belloni E., Ardito R., Braghin F., Corigliano A. (2017). Mechanical low-frequency filter via modes separation in 3D periodic structures. Appl. Phys. Lett..

[15] Park S., Jeon W. (2021). Ultra-wide low-frequency band gap in a tapered phononic beam. J. Sound. Vib..

[16] Gu C., Ma L., Ou Z., Lei F. (2024). Propagation characteristics of lamb waves in a functionally graded material plate with periodic gratings. Mech. Adv. Mater. Struct..

[17] Man X.f., Xia B.z., Luo Z., Liu J. (2019). 3D Hilbert fractal acoustic metamaterials: Low-frequency and multi-band sound insulation. J. Phys. D Appl. Phys..

[18] Chen Z., Chong Y.B., Lim K.M., Lee H.P. (2024). Reconfigurable 3D printed acoustic metamaterial chamber for sound insulation. Int. J. Mech. Sci..

[19] Sun Y., Zhang G., Lee H.P., Zheng H., Luo Z., Li F. (2024). Sound transmission of truss-based X-shaped inertial amplification metamaterial double panels. Int. J. Mech. Sci..

[20] Jiang Z., Zhou Y., Zheng S., Liu J., Xia B. (2023). Waveguides induced by replacing defects in phononic crystal. Int. J. Mech. Sci..

[21] Lekhal D., Mekkakia-Maaza N.E., Lakhdari A. (2021). Finite element analysis of surface elastic waveguide based on pyramidal phononic crystal. Micro Nano Lett..

[22] Zheng Y., Wang C., Tan Z., Zhang Z., Jiang J., Cheng B., Cao J. (2023). Realization of a novel multimode waveguide with photonic band gap hopping based on coupled-resonator optical waveguide theory. Results Phys..

[23] He Z., Zhang G., Chen X., Cong Y., Gu S., Hong J. (2023). Elastic wave harvesting in piezoelectric-defect-introduced phononic crystal microplates. Int. J. Mech. Sci..

[24] Gantasala S., Thomas T., Rajagopal P. (2023). Enhanced piezoelectric energy harvesting based on sandwiched phononic crystal with embedded spheres. Phys. Scr..

[25] Zhao L., Lu Z., Ding H., Chen L. (2024). A viscoelastic metamaterial beam for integrated vibration isolation and energy harvesting. Appl. Math. Mech..

[26] Kushwaha M.S., Halevi P., Dobrzynski L., Djafari-Rouhani B. (1993). Acoustic band structure of periodic elastic composites. Phys. Rev. Lett..

[27] Liu Z., Zhang X., Mao Y., Zhu Y.Y., Yang Z., Chan C.T., Sheng P. (2000). Locally Resonant Sonic Materials. Science.

[28] Li J., Yang P., Li S. (2020). Phononic band gaps by inertial amplification mechanisms in periodic composite sandwich beam with lattice truss cores. Compos. Struct..

[29] Iqbal M., Jaya M.M., Savadkoohi A.T., Baguet S. (2025). Vibration attenuation of dual periodic pipelines using interconnected vibration absorbers. Eng. Struct..

[30] Wang C., Yao X., Wu G., Tang L. (2021). Complete vibration band gap characteristics of two-dimensional periodic grid structures. Compos. Struct..

[31] Geng Q., Kong L., Yang X., Shao Z., Li Y. (2023). Phononic crystal pipe with periodically attached sleeves for vibration suppression. Int. J. Mech. Sci..

[32] Liu W., Chen J., Liu Y., Su X. (2012). Effect of interface/surface stress on the elastic wave band structure of two-dimensional phononic crystals. Phys. Lett. A.

[33] Panahi E., Hosseinkhani A., Frangi A., Younesian D., Zega V. (2022). A novel low-frequency multi-bandgaps metaplate: Genetic algorithm based optimization and experimental validation. Mech. Syst. Signal Process.

[34] Pereira J., Ruiz R.O. (2025). Multi-objective functions for the optimization of piezoelectric metamaterials under pre-established bandwidth excitations. Mech. Syst. Signal Process.

[35] Haghpanah B., Ebrahimi H., Mousanezhad D., Hopkins J., Vaziri A. (2016). Programmable Elastic Metamaterials. Adv. Eng. Mater..

[36] Xin Y.J., Huang R.N., Li P., Yan H., Dong X.J., Yan Q., Sun Y.T., Cheng S.L., Zhao Q.X. (2023). Labyrinth acoustic metamaterials with fractal structure based on Hilbert curve. Phys. B Condens. Matter.

[37] Oftadeh R., Haghpanah B., Vella D., Boudaoud A., Vaziri A. (2014). Optimal Fractal-Like Hierarchical Honeycombs. Phys. Rev. Lett..

[38] Man X., Luo Z., Liu J., Xia B. (2019). Hilbert fractal acoustic metamaterials with negative mass density and bulk modulus on subwavelength scale. Mater. Des..

[39] Wang K., Liu Y., Liang T. (2016). Band structures in Sierpinski triangle fractal porous phononic crystals. Phys. B Condens. Matter.

[40] Cheng S.L., Yang H.Y., Ding Q., Yan Q., Sun Y.T., Xin Y.J., Wang L. (2022). Low Frequency Band Gap and Wave Propagation Properties of a Novel Fractal Hybrid Metamaterial. Phys. Status Solidi B.

[41] Du Q., Zeng Y., Xu Y., Yang H., Zeng Z. (2018). H-fractal seismic metamaterial with broadband low-frequency bandgaps. J. Phys. D Appl. Phys..

[42] Zhang C., Chen X., Dong T., Hao T., Wang J. (2024). Study of Fractal Honeycomb Structural Mechanics Metamaterial Vibration Bandgap Characteristics. J. Vib. Eng. Technol..

[43] Yang S., Yin J.H., Zhu X.J., Wang K., Zhang S.k., Cao L., Guo P.Y., Liu Y. (2025). Investigation and optimal design of band gap tunability in fractal phononic crystals. Acta Acust..

[44] Ribeiro L.H.M.S., Dal Poggetto V.F., Beli D., Fabro A.T., Arruda J.R.F. (2022). Investigating the stochastic dispersion of 2D engineered frame structures under symmetry of variability. J. Sound. Vib..

[45] Wang L., Wang Z., Lu X., Shi L. (2023). Novel applications of local optimization semi-Cartesian grid for the complex band structure analysis of phononic crystals. Appl. Math. Model..

[46] Ma Y.K., Guo W., Cui Y.M., Wang Y.F., Laude V., Wang Y.S. (2025). Attenuation of Lamb waves in coupled-resonator viscoelastic waveguide. Int. J. Mech. Sci..

[47] Lakes R. (1987). Foam Structures with a Negative Poisson’s Ratio. Science.

[48] Du C., Wang Y., Kang Z. (2023). Auxetic Kirigami Metamaterials upon Large Stretching. ACS Appl. Mater. Interfaces.

[49] Bertoldi K., Reis P.M., Willshaw S., Mullin T. (2010). Negative Poisson’s Ratio Behavior Induced by an Elastic Instability. Adv. Mater..

[50] Novak N., Mauko A., Ulbin M., Krstulović-Opara L., Ren Z., Vesenjak M. (2022). Development and characterisation of novel three-dimensional axisymmetric chiral auxetic structures. J. Mater. Res. Technol..

[51] Gibson L.J., Ashby M.F. (1982). The Mechanics of Two-Dimensional Cellular Materials. Proc. R. Soc. Lond..

[52] Wojciechowski K.W. (1987). Constant thermodynamic tension Monte Carlo studies of elastic properties of a two-dimensional system of hard cyclic hexamers. Mol. Phys..

[53] Wojciechowski K.W. (1989). Two-dimensional isotropic system with a negative poisson ratio. Phys. Lett. A.

[54] Evans K.E. (1991). Auxetic polymers: A new range of materials. Endeavour.

[55] Milton G.W. (1992). Composite materials with poisson’s ratios close to—1. J. Mech. Phys. Solids.

[56] Ting T.C.T., Chen T. (2005). Poisson’s ratio for anisotropic elastic materials can have no bounds. Q. J. Mech. Appl. Math..

[57] Greaves G.N., Greer A.L., Lakes R.S., Rouxel T. (2011). Poisson’s ratio and modern materials. Nat. Mater..

[58] Lakes R.S., Huey B. (2023). Poisson’s Ratio beyond the Classically Allowable Range in Chiral Isotropic Elastic Materials: Effect of *k* and Experiment. Phys. Status Solidi B.

[59] Alderson A., Wojciechowski K.W. (2005). Preface: Auxetic materials and anomalous systems. Phys. Status Solidi B.

[60] Han S., Han Q., Ma N., Li C. (2023). Design and reinforcement-learning optimization of re-entrant cellular metamaterials. Thin-Walled Struct..

[61] Hu Y., Li Y., Zhang Y., Ding S., Wang R., Xia R. (2024). Design methodology for functional gradient star-shaped honeycomb with enhanced impact resistance and energy absorption. Mater. Today Commun..

[62] Liu M., Cao Y., Sun D.Q., Nie C.R., Wang Z.J. (2024). In-Plane Dynamic Cushioning Performance of Concave Hexagonal Honeycomb Cores. Shock Vib..

[63] Jin E., Lee I.S., Kim D., Lee H., Jang W.D., Lah M.S., Min S.K., Choe W. (2019). Metal-organic framework based on hinged cube tessellation as transformable mechanical metamaterial. Sci. Adv..

[64] Lim T.C. (2019). An Anisotropic Auxetic 2D Metamaterial Based on Sliding Microstructural Mechanism. Materials.

[65] Pozniak A.A., Wojciechowski K.W. (2014). Poisson’s ratio of rectangular anti-chiral structures with size dispersion of circular nodes. Phys. Status Solidi B Basic Solid State Phys..

[66] Mizzi L., Attard D., Gatt R., Pozniak A.A., Wojciechowski K.W., Grima J.N. (2015). Influence of translational disorder on the mechanical properties of hexachiral honeycomb systems. Compos. Part B Eng..

